# Strengthening Equity and Inclusion in Urban Greenspace: Interrogating the Moral Management & Policing of 2SLGBTQ+ Communities in Toronto Parks

**DOI:** 10.3390/ijerph192315505

**Published:** 2022-11-23

**Authors:** Claire Davis, Sara Edge

**Affiliations:** Department of Geography and Environmental Studies, Toronto Metropolitan University, Toronto, ON M5B 2K3, Canada

**Keywords:** greenspace, parks, queer, sexuality, public space, policing, environmental justice, sustainability, marginalized communities, moral control

## Abstract

There is growing recognition that greenspace provides invaluable benefits to health and wellbeing, and is essential infrastructure for promoting both social and environmental sustainability in urban settings. This paper contributes towards efforts to build ‘just’ and equitable urban sustainability, and more specifically greenspace management, by drawing attention to hostility and exclusion experienced by two-spirit, lesbian, gay, bisexual, transgender, queer, genderqueer, pansexual, transsexual, intersex and gender-variant (2SLGBTQ+) park occupants. There is evidence that access to greenspace is inequitable—despite ongoing media accounts of targeted violence and discriminatory police patrolling of 2SLGBTQ+ communities in urban parks, this population has not received adequate research attention. This paper examines systemic barriers that impede urban greenspace access among 2SLGBTQ+ communities, including how the threat of violence in greenspace limits opportunities for accessing benefits associated with naturalized settings. These themes are explored within the context of the City of Toronto, Canada. Our mixed-method approach draws upon key informant interviews, key document content analysis, and ground-truthing. Our findings reveal how queer corporeality, kinship and love subvert deeply entrenched heteronormative social values and understandings of sexuality, partnership, gender, and use of public space, challenging institutional understandings of morality and daily life. The paper concludes by reflecting on the state of 2SLGBTQ+ communities’ relationships to greenspace, and potential ways forward in building greater inclusivity into the social fabric of park design and management.

## 1. Introduction

Parks and urban greenspace are vital resources to sustainable cities and critical environmental determinants of health [1]. Urban greenspace helps regulate pollution, mitigate urban heat island effects, relieve stress, and encourage food access, social interaction, physical activity, and general wellbeing [2,3]. Activists and scholars have called upon governments and institutions to radically reframe urban sustainability discourse to extend beyond the limited dominant narrative of protecting natural resources (such as water, trees, etc.), and to ask what, and whom, sustainability is for [4,5]. It is increasingly accepted that “social justice is a necessary condition for sustainability, not vice versa” [6]. Therefore a ‘just’ sustainability approach requires ensuring that marginalized populations have equitable and inclusive access to resources including land, water, and health related services [5].

Greenspace access is increasingly being recognized as an environmental justice issue, due to growing recognition of barriers due to underlying socio-spatial inequalities [1]. Environmental injustices arise when certain populations (based on their identity, appearance, or lived experience) face restricted access to environmental benefits and/or disproportionate exposure to environmental hazards [7]. Greenspace remains an under-explored topic in environmental justice research, with understandings of associated adverse impacts upon affected populations remaining limited, along with discussions around appropriate remedial action from a greenspace planning and management perspective [8,9]. Further, knowledge on greenspace use, access barriers and related injustices experienced by 2SLGBTQ+ communities is particularly lacking in environmental scholarship despite historical and contemporary media documentation of urban parks being sites of violent homophobic attacks [10,11], queer-targeted police sting operations [12], and other forms of exclusion. 

There is a need for a better understanding of the systemic barriers that impede urban greenspace access among 2SLGBTQ+ communities. This includes understanding how the threat of violence in greenspace limits social interaction for 2SLGBTQ+ identifying people, and restricts opportunities for accessing benefits associated with urban parks and naturalized settings [8,13]. Further, little attention has been given to how and whether institutions responsible for urban sustainability and greenspace management are considering issues of access for 2SLGBTQ+ communities. It is imperative to interrogate how heterosexuality and associated normative behaviour is reproduced and made privileged in public greenspace through regulation, enforcement, intimidation and other signalling of ‘appropriate conduct’, in addition to related impacts upon the ability of those with marginalized sexual and gender identities to access health-supporting greenspace environments. 

Accordingly, this paper explores perceived challenges, concerns and experiences of exclusion among 2SLGBTQ+ communities in Toronto’s urban greenspace based on their non-normative sexual and gender identities. The specific objectives are to examine what is known about the experiences of exclusion/access for 2SLGBTQ+ communities in urban greenspace from the perspective of diverse stakeholders, and analyze whether and how these concerns are being addressed within the City of Toronto’s urban greenspace and park management practice. 

In this paper, greenspace refers to public parks and does not include spaces such as brownfields, highway underpasses, cemeteries, or vegetated land that is not designated public park space. Additionally, within the context of this research, 2SLGBTQ+ and queer are used interchangeably as umbrella identifiers for two-spirit, lesbian, gay, bisexual, transgender, pansexual, transsexual, intersex and gender-variant individuals. 

We begin by integrating insights from the fields of environmental justice, moral geography, urban political ecology, and queer theory to establish a novel framework for understanding the complex interchange between urban greenspace access, management, morality and justice. We then outline our methodological approach, followed by a presentation of key findings. The discussion situates these findings within existing literature, concluding with considerations for future research and thoughts on strengthening urban sustainability through equitable access.

## 2. Literature Review

### 2.1. Greenspace Access as an Environmental Determinant of Health

“Greenspace” provides various direct and indirect human health benefits [2], including promoting physical activities/exercise [14], reducing negative emotions, supporting higher energy levels and attention spans, and overall feelings of wellbeing [9,15]. Additionally urban greenspace provides land for growing food, and helps to mitigate pollution and urban heat island effects [2]. Many greenspace benefits are “non-material” and difficult to quantify. Limited attention has been given to urban greenspace’s cultural, spiritual, and social values, despite recognition that these are no less important to wellbeing [2]. 

### 2.2. Greenspace Access & Socio-Environmental Justice

A growing number of studies have demonstrated that social inequities are associated with poor urban greenspace access. For example, studies from cities in Canada [16], China [17] and Australia [18] that all utilized geospatial analytical techniques have consistently demonstrated that low-income populations are concentrated in areas with poor greenspace availability. Yang et al. [17] further specify that older adults, immigrants, and those with lower education are among those disproportionately experiencing limited proximity to greenspace. Further, Sharif et al. [18] indicate that the relocation and/or forced displacement of low-income groups over time due to affordability challenges is also associated with an even further decline in greenspace access. From this standpoint, inequity in urban greenspace access threatens the development of sustainable cities through undermining public health and resident wellbeing [17].

Access to greenspace is increasingly acknowledged as an environmental justice issue [2,3,8,19]. Environmental injustices arise when certain populations (based on their identity, appearance, or lived experience with poverty) face restricted access to environmental benefits and/or disproportionate exposure to environmental hazards [19]. Most studies that have examined greenspace from an environmental justice lens have focused on particular ethnic or racial groups [20,21]. Brownlow [22] discusses the fear of violence in parks expressed by racialized citizens in Philadelphia, Pennsylvania to explore relationships between power, control, and access. Byrne and Wolch also discuss the racial politics of parks, asserting that park development reflects hegemonic “ideologies of land use, histories of property development, planning philosophy and the spatial expression of racial discrimination” [13] (p. 753). Studies across several global cities have connected social identities to spatial injustices related to accessing “quality park space” [8].

While awareness of the inequalities and related justice implications in accessing urban greenspace is growing, specific focus on the experiences of 2SLGBTQ+ individuals and communities remains absent. Further, 2SLGBTQ+ communities already experience inequitable access to public spaces and social services in general, and therefore experiences of deprivation and exclusion are cumulative [23,24].

### 2.3. Urban Parks and Greenspace as Political & Moral Landscapes

Urban political ecology literature reveals how urban parks operate as moral spaces, and how ‘disruption’ to what is perceived as normative moral behaviour in these spaces is institutionally and socially challenged and regulated. While parks are framed as ‘wild’ urban spaces, they are in fact highly managed and politically motivated [25]. Catungal and McCann’s study of Stanley Park, Vancouver, British Columbia, a known gay cruising area, examined expressions of love, intimacy and sexuality in park space through a governance lens [26]. They argue that “visible transgressions”—(i.e., expressions of non-normative sexual identities)—act to destabilize hegemonic norms or predominant cultural expectations, behaviours and identities, including presumptions around and/or acceptance of heterosexual forms of intimacy or affection, and/or preference for nuclear families, etc.). They explore how greenspace uses and users are consequently managed, included or excluded through social norms, planning, regulatory and policing processes that collectively reinforce heteronormative behaviours and expectations. The researchers conclude that the ways in which the state reacts to the disruption of heteronormative norms, reveals how the foundation of order is “social, power-laden and uneven” [26]. 

Post-structuralist critiques in urban political ecology stress the need to engage with politics and discourse when examining and managing environmental amenities. Grove discusses this in terms of nature in the city, noting “struggles over meanings and practices of nature and the city shape identities that make some forms of urban metabolisms possible while foreclosing others” [27] (p. 209). Grove positions nature in the city as a social creation rather than existing devoid of political input. Such an understanding creates space for critiquing the social and geographical management and use of urban parks. While greenspace planning happens at an institutional level, urban political ecology identifies that formal public and private governance systems are also subverted and challenged by individuals and communities in their everyday lives [28]. In this way, the material conditions and socio-political landscapes of urban parks and greenspaces are impacted by how these environments are imagined through a diverse array of users [9]. 

## 3. Materials and Methods

This exploratory study, approved by Toronto Metropolitan University Research Ethics Board (REB 2020-422), draws upon mixed-qualitative methods and a social constructionist approach [29], enabling a deep dive into ascribed meanings expressed by study participants, and thick description of emerging themes [30,31]. 

### 3.1. Study Context

The City of Toronto is home to 2.9 million people and is one of the most multicultural cities in the world [32]. As of 2016, 5% of Toronto adults 18 years and over self-identified as “homosexual” or “bisexual”, enough to populate a large city in and of itself [33]. In terms of gender identity, it is estimated that 1 in 200 adults identify as non-cisgender. However, there is no official data on the proportion of transgender, non-binary, genderqueer and gender non-conforming people living in Toronto [33]. Toronto hosts one of the largest Pride festivals in the world each June, with over a million attendees [34]. In Ward 13 Church-Wellesley Village, there is a 2SLGBTQ+ enclave informally referred to as the Gay Village, where many queer-oriented businesses, social spaces and 2SLGBTQ+ identifying people reside [35]. 

While Toronto’s queer communities are prominent, discrimination and targeted violence persist. As recently as June 2021, David Gomez, a queer man, was approached and threatened by a small group while leaving Hanlan’s Point Park beach. Members of the group accosted Gomez with homophobic slurs, before ‘nearly beating him to death’, and leaving him unconscious [10]. 

Throughout the Fall of 2016, there was considerable coverage in Toronto news media around a 6-week long undercover police sting operation called Project Marie, which occurred in Marie Curtis Park [12]. Dressed in plainclothes, male police officers combed the park and approached men, soliciting sexual encounters. By the end of the operation, the police had filed 89 charges against 72 people with only one criminal charge laid, with most consisting of bylaw infractions, like trespassing [12]. Toronto-based queer media outlet Xtra argued that the significant time and resource investments made for such minor infractions was indicative of “underlying homophobic motivations” as opposed to public safety. They suggested that having plainclothes officers approach men and solicit them for sex in the park was entrapment [36]. This view was supported by 2SLGBTQ+ City Counsellor Kristyn Wong-Tam, who called for City Council to “reconvene the Community Advisory Committee on Lesbian, Gay, Bisexual, and Transgender Issues to help inform how the City conducts its business and deploys its resources affecting this minority population” [37]. Member of Provincial Parliament Cheri DiNovo similarly wrote an open letter to Ontario’s Attorney General stating: “An undercover sting operation should be for serious crimes, not for intimidating and harassing gay men and trans people who are meeting each other in public spaces” [38]. The Toronto Police never issued an apology for the raid.

### 3.2. Key Informant Interviews

Open-ended key informant interviews were conducted virtually, with park management staff, city planners, politicians and 2SLGBTQ+ community activists who had organized around park access and policing. All participants were 18 years of age or older and have experience (1) working for organizations (including non-profits, government, etc.) that provide services, legal representation and/or counselling to LGBTQ2S+ individuals, (2) professional involvement in Toronto park planning, management or policing operations and/or (3) a current or previous history of experience with activism and organizing around the issue of accessibility and/or park sting operations. Key informants were identified and contacted through publicly accessible venues and in some circumstances through snowball sampling techniques. There were no incentives provided for participation. All interviews were 60–90 min in length, audio-recorded and transcribed verbatim. Interviews sought to understand greenspace experiences, the motivations and actions of various stakeholder groups involved in park access, and their respective perspectives on what is considered appropriate use of public greenspace, along with views on institutional management and regulation of these standards. 

Participants were interviewed in both their professional and personal capacities to illustrate the juncture between individual’s perceptions and potential influence on institutional practice and the impact of normative or regulatory institutional practices. Interestingly, key informants (KIs) all identified as 2SLGBTQ+ although this was not intended in the research design. A range of stakeholders and practitioners who were similarly positioned were approached to participate, with many declining the opportunity to be interviewed due to a perceived lack of expertise. This raises interesting questions around levels of awareness, perceived responsibility and advocacy within fields of environmental management, sustainability and/or planning, particularly among stakeholders and decision-makers that do not directly identify with the 2SLGBTQ+ community. Other intended key informants included members of the Toronto Police Services, including the LGBT Liaison Officer. This group was unresponsive to multiple inquiries, potentially in fear of backlash in light of recent global attention on police misconduct and brutality. 

The first author, who conducted the interviews, was conscious of power relations throughout the interview process. Their queer identity allowed for interactions with activists and community members to be carried out in a more conversational, peer-to-peer manner, helping to establish rapport. As one participant noted, “I was nervous for the interview, but now I see you are one of us.” This promoted a safe(r) relational space for informants to reflect and share their perceptions and experiences. The interviewer was conscious of their use of terminology, allowing participants to use their own diction, especially around sexuality and gender identities, adjusting verbiage as interviews went on in flex with participants’ self-determination. For example, if a key informant referred to themselves as a trans woman, the researcher would then refer to them as a trans woman rather than an umbrella term such as “2SLGBTQ+ person”. Inductive and deductive coding unfolded iteratively in collaboration with the second author, where coding and interpretations were compared and contrasted in order to arrive at consensus [39]. 

### 3.3. Key Document Content Analysis

Key document analysis is a tool that can be used to make social practices, normative expectations, discursive framings and institutional standards of morality and heteronormativity explicit. Text is extracted from key documents through quotations, excerpts and passages, which are then organized and analyzed to identify convergence and corroboration across different data sources [40]. The approach involves examining and critiquing language and discourse to identify how topics of queerness and marginalization are either engaged with or avoided altogether (see Table 1 for Key Documents analyzed).

### 3.4. Ground-Truthing

Ground-truthing, through on-the-ground observations of space, has become commonly applied in broader geographic and social research to verify data in everyday contexts, and understand biases and perspectives presented as truth [41]. Instances of in/exclusion or othering in greenspace were examined through field observations of symbolic and concrete codification of prescribed desirable/undesirable identities and behaviours of greenspace users. Symbolism can evoke particular emotions, responses, and understandings [42]. For example, Pride flags may indicate the inclusion and representation of 2SLGBTQ+ communities. This could, in turn, have an overt or subtle influence on the power dynamics present in public greenspace where queer people are visually cued to belong and recognized as valued community members. As Lombardo & Meier explain, “The choice of public symbols can purposely reproduce or counteract existing power relations.” [42] (p. 327). We photographically documented signage, infrastructure/landscape design, and instances of public interference or expression. This promoted further understanding of interviewees’ perceptions of greenspace, and forms of inclusion/exclusion. Field observations also helped to confirm or challenge that which was stated within key planning and management documents. 

## 4. Results

### 4.1. Morality in Greenspace Management

The City of Toronto Municipal Code Chapter 608: Parks outlines a lengthy list of activities and behaviours that are permissible in Toronto greenspace, as well as activities that are allowed only with a permit, such as large organized group gatherings [43]. The Municipal Code stipulates that “While in a park, no person shall create a nuisance by loitering, spying, accosting, frightening, annoying or otherwise disturbing other persons” [43]. Key informants expressed confusion and even suspicion when discussing the anti-loitering bylaws in the Municipal Code, citing the vague language may be intentional, providing greater reign when policing specific communities in the name of upholding the comfort and morals of others. As one planner shared:

I think one of the things in the municipal code is ‘any use of a park or public space that infringes on the enjoyment of another person’ [is an infraction]. Which is so problematic to me because, then you know you’re again deciding who’s enjoyment and use is correct and takes precedence over someone else’s enjoyment and use of a public space. And we see how that very kind of innocuous seeming language is kind of weaponized in these situations.

(Environmental Planner)

Toronto’s Municipal Code for parks details conduct regulations that overtly implicate sexuality, morality and the use of public greenspace. It states: 

A. While in a park, no person shall:

(5) Engage in any form of sexual behaviour; or 

(6) Be nude. 

B. For the purposes of Subsection A (6), a person is nude who is clad as to offend against public decency or order.

A politician key informant explained how the regulation of sexuality in greenspace is part of a broader, deeply entrenched “anti-sex outlook”, that disproportionately targets 2SLGBTQ+ communities over heterosexual counterparts when interpreting anti-sex values expressed in the municipal code. Heterosexual couples kissing is seen as an iconic, cinematic and celebrated scene to witness in greenspace, while queer people engaging in the same activities are often vilified or criminally sanctioned.

I mean you know, for as long as humanity has existed parks and public spaces have been places for heterosexuals to meet, right? And you know, we can all think of pictures, you know in Paris of heterosexual couples kissing on the you know banks. Well you know why is it only okay for heterosexual couples to do that to have obvious you know sexual desire for each other in a public space?

(Politician)

While police sting operations, such as Project Marie, and fears of queer sexual activity in greenspace orbit around more sensationalized discussions of public sex, this reflection reminds us that very common displays of affection and love are widely accepted for heterosexual couples. Yet, the same behaviours, when visibly queer, are rejected.

### 4.2. Policing & Exclusion of 2SLGBTQ+ Communities in Greenspace

A recurring theme impacting 2SLGBTQ+ greenspace access was the negative relationship between the police force and queer communities in greenspace. A local politician with decades of experience advocating for queer rights explained how the threat that police presence brings to queer occupation of greenspace is an ongoing and ceaseless issue:

I mean it was always policed, there were always police and there were always arrests. So that was the shadow hanging over the use of parks.

(Politician)

Another key informant referenced the police sting operation that occurred in Marie Curtis Park in 2016 as a catalyst for renewed attention around the issue:

I think about what happened in Marie Curtis park a couple of years ago. That wildly disproportionate sting operation that I don’t think resulted in any criminal charges. It was just a horrible display of homophobia by the Toronto police […] it brought up again, the sort of really bad history between the police and the queer community.

(Queer Activist)

The City of Toronto’s Municipal Code, Chapter 608-53: Enforcement outlines the power police officers have over greenspace conduct and the process that follows infractions:

- Any officer is authorized to inform a person of the provisions of this chapter and request compliance with it.
- Any officer is authorized to order a person believed by the officer to be contravening or who has contravened any provision of this chapter to:
  (1) Stop the activity constituting or contributing to the contravention;
  (2) Remove from the park to a pound or storage facility any animal or thing owned by or in control of the person who the officer believes is or was involved in the contravention; or
  (3) Leave the park.

The vague language leaves much power to the police on deciding when a bylaw has been contravened. It also states that the main method of managing ‘problematic’ behaviour is to request the individuals causing disruption to vacate the park, rather than issuing an arrest. Yet, sting operations targeting queer communities have resulted in instances of criminalization. Multiple key informants with lived experience expressed fear of arrest, indicating that police presence incites fear and abuse of power, rather than promoting safety, and is incongruent with the city’s framing of natural spaces as inclusive sites for leisure and refuge:

I’m scared of the police for sure, because I don’t trust them. And I never will, never have. Police are so out of place in a park.

(Queer Activist)

Policing… doesn’t have a place in public park space, it seems to me.

(Politician)

This same informant elaborated how there are discrepancies in policing and enforcement where heterosexual couples displaying affection, love and sexuality in greenspace is seen as socially acceptable, whereas queer love and affection is something to intercept and criminalize:

You know it’s sort of borderline okay if it’s heterosexual. But it’s absolutely not okay if it’s homosexual. So I mean I think there’s the fact that when we’ve used it the same way that straight people have used public spaces, it’s been criminalized in a way that heterosexuals using that space is not—I mean what is a cop’s reaction to kids making out, you know, in a public space on a beach let’s say? […] The difference between them and two men making out would be an arrest and still is apparently, right?

(Politician)

These findings suggest greenspace management and enforcement practice must better consider how to “signal” to 2SLGBTQ+ people that greenspace is a safe space to occupy.

### 4.3. 2SLGBTQ+ Inclusion in Greenspace Accessibility Policy & Practice

Toronto’s Parkland Strategy [44] and Parks Plan [45] outline the city’s park planning operations, including enhancing and expanding existing parks. These plans are overseen by the General Manager of Parks, Forestry and Recreation, the Acting Chief Planner and the Executive Director of City Planning and are shaped by the policies in the City of Toronto’s Official Plan. These documents were examined to consider how issues of accessibility and inclusion are considered. For example, the Parkland Strategy Final Report states that “The demographic composition of neighbourhoods is also changing, as are park user preferences and expectations.” [44] (p. 30). This indicates an awareness of changing demographics and needs. However, beyond acknowledging diverse access needs in a general way, municipal documents lack specific action plans for ensuring equitable access beyond physical and spatial barrier reductions (e.g., ramps, physically accessible washrooms). There is no discussion around specific marginalized identities and how inaccessibility uniquely manifests within and across these communities. 

A guiding principle in the Parkland Strategy is to “Include everyone by removing barriers so that parks and other open spaces are inclusive and inviting places that are equitably accessible for people of all ages, cultures, genders, abilities, and incomes” [44] (p. 8). This statement is not accompanied by any further reflection on unique barriers for different populations, or the ways in which certain populations may be (un)intentionally excluded. The mention of gender indicates there is room for expanding upon notions of gender and acknowledging queerness as an identity with specific needs in greenspace, especially pertaining to the use of washrooms. However, there is an apparent disconnect between more inclusive language in relation to genders in overarching strategic documents and the binaried language that remains within the official Municipal Code or on-the-ground greenspace facilities. During interviews, key informants were asked to describe what accessibility means to them, how or whether accessibility is considered in greenspace management, and how Toronto fares in ensuring equitable access to greenspace. Two queer planners shared similar perspectives, each expressing that comprehensive understandings of accessibility have yet to be adequately institutionalized:

I think Toronto definitely is lacking in sort of the quality of accessibility that we have within the city. And that is to greenspace and transportation networks. I know there’s definitely things in the works to improve that for sure, but I think we should take it even further.

(Urban Planner)

They added taking it “even further” means considering concerns beyond physical and spatial barriers and considering what access means across several marginalized communities. Another environmental planner similarly argued that greenspace access considerations tend to fall short when it comes to cultural limitations including a lack of regard for sexuality and access for queer and marginalized communities:

[We must consider]… whether the design and the amenities, and the programming in that space are ones that meet your needs. So that could be a physical accessibility limitation, there’s lots of those in parks. But it could also be, you know, like a cultural limitation as well…And, so when I think about accessibility, I think of those different levels.

(Environmental Planner)

A key informant with a history of activism around queer use of greenspace concurred that language around “barrier-free” access planning is often limited to a focus on physical elements:

They don’t often think about accessibility in terms of sort of cultural or even social elements around who feels you know… welcome in a space or feeling like you know there’s something there that reflects the person, who you are and how you want to use the space.

(Queer Activist)

One planner suggested that although greenspace is framed to be “neutral” and “democratic”, they perceive some access barriers to be intentional within design and management practices:

What can happen in that space and who is meant to use it and what is the correct way of using a park or a public space and what is an incorrect way? I think, you know, people always talk about parks as these democratic spaces that are open for everyone, and you know, sometimes they’re talked about as neutral spaces in our cities and I think the more I’ve learned about parks and the more I’ve worked in this area, I realized that that’s that’s not true at all. They’re definitely not neutral spaces, they’re highly governed and they’re not open to everyone and that’s by design.

(Environmental Planner)

In the Toronto Parks and Recreation Facilities Master Plan, there is an entire section focused on “Accessibility for All” [46]. Therein it states that greenspaces are “integral and visible elements of the public realm” in Toronto and “must be truly accessible and inclusive to be effective.” Throughout the Master Plan there are discussions of improving accessibility through “convenience and fairness in access to resources” to provide a quality experience [46]. The idealism and lack of specificity when it comes to outlining targeted goals and implementation strategies in these documents becomes clear when the only notable action item following these statements is to review the permitting process for large group gatherings in greenspace to ensure they, and other amenities, are affordable.

The perspectives shared by key informants that have worked with planning bodies throughout their careers in Toronto indicated that while a more fulsome definition of accessibility is a goal, this has not yet institutionalized or become implemented through design and planning processes:

I think there are lots of really great people who are thinking this way already. I don’t think it’s institutionalized in a city setting in terms of how we plan and operate parks, but you know there’s a lot of really amazing advocates and urban thinkers I think that are already kind of raising this. We need to think about the social as much as we need to think about or even more than we need to think about the physical because that’s such an important element of inclusion, equity and access.

(Urban Planner)

**Figure 1:**
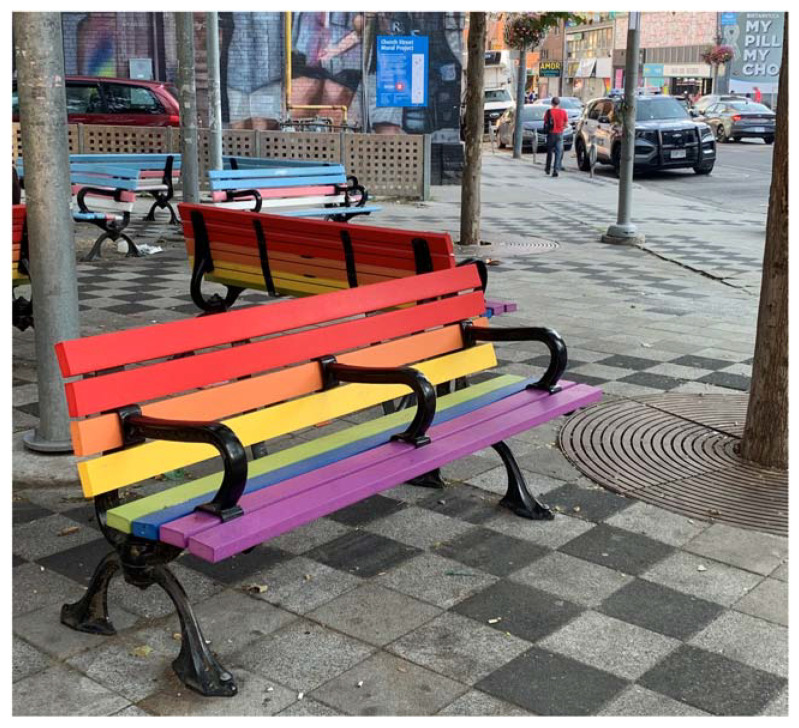
Pride flag painted benches situated along the west side of Barbara Hall park in Toronto’s Church-Wellesley Village neighbourhood.

In addition to what is enshrined (or neglected) in policy, multiple participants referred to the importance of symbolism at the ground level of lived experience, to creating inclusive, accessible park spaces, with specific attention drawn to the use of Pride and Trans flags (as seen in Figure 1). In the words of one respondent:

I think one of the aspects of public parks generally that could be changed is just to mark them as queer positive spaces, to have a pride symbol there and say this is a queer positive space and that signals to people who are going there looking to you know, like beat up queers or, like target queers, it signals to them that they’re not welcome doing that there, it makes it safer and welcoming to queers.

(Urban Planner)

**Figure 2:**
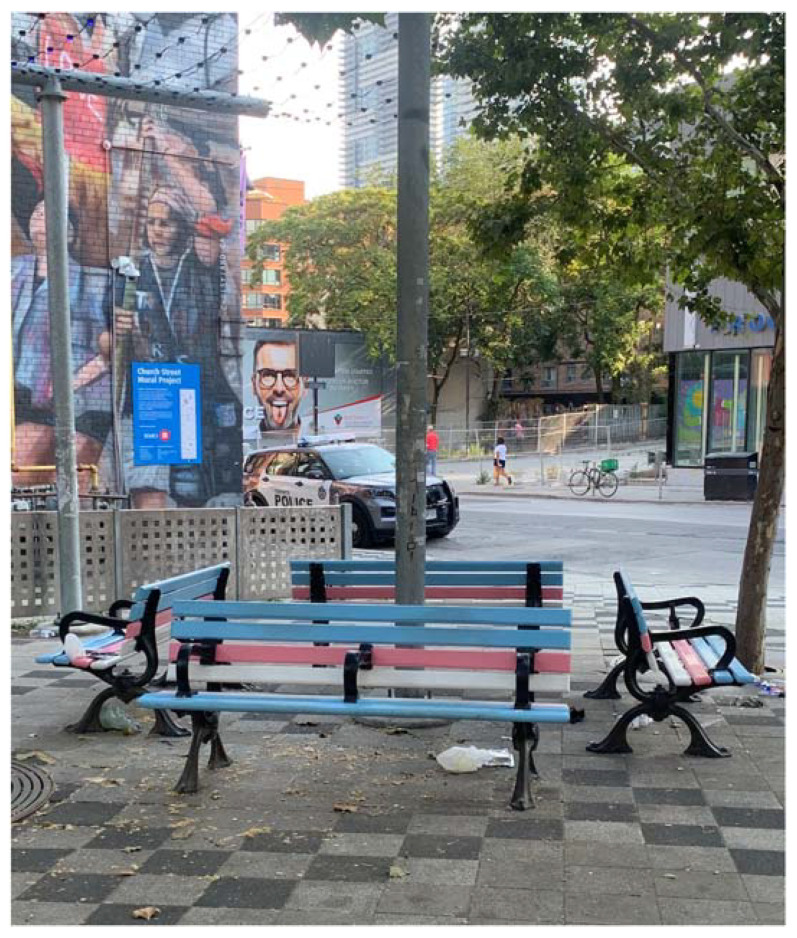
Trans flag benches located in Barbara Hall Park. A police cruiser can be seen parked on the perimeter of the park.

Through ground-truthing, Trans and Pride flag-painted benches (see Figure 1 and Figure 2) were located and documented in Barbara Hall park, situated in Toronto’s “Gay Village” neighbourhood. Notably, a police cruiser can also be seen in the background of the photographs, parked on the street that lines the park. A key informant who was involved in creating some of the flag symbolism in Barbara Hall Park reflected on how they felt contradiction when painting the benches (in the name of supporting one marginalized group), upon infrastructure that is simultaneously anti-homeless (i.e., arm-rests in place to prevent sleeping), adding that they chose to proceed because it was a step in the right direction:

So we put some trans and rainbow benches in that park. You know the rainbow crosswalks, trans crosswalks, I was kind of part of the group that did some of that. So these are the symbols, right? So it’s a good way to say you are welcome, it’s clear, it’s coded, it’s feasible. But it’s kinda like pride [parades/celebrations], when people are still hungry and homeless the day after. So what about the day after, when people are still sleeping in parks? I find myself making contradictions in my life, trying to make a difference. But the benches do seem like a good symbolic thing.

(Queer Activist)

It can be gathered from this reflection that the greenspace benches are symbols, in this particular context, of both acceptance and exclusion. The representative flags may intend to signal that 2SLGBTQ+ communities have a place in greenspace, yet there is still inadequate consideration for the intersections of multiple forms of social marginalization (particularly given the queer community is disproportionately represented among homeless populations). This also evokes further thoughts around morality and which aspects included in the lived realities of queer life are acknowledged and publicly accepted.

### 4.4. Perspectives on Strengthening Equity in Sustainable Greenspace Management

Informants expressed differing opinions and views on how greenspace management should ideally move forward, along with who should be responsible for ensuring 2SLGBTQ+ inclusion, access, and safety in Toronto greenspace. Multiple key informants indicated that they would like to see community ambassadors that are trained crisis responders rather than uniformed police monitoring public parks, questioning whether police enforcement truly promotes community safety. Many believed replacing police presence with community-based safety response measures would help address fears of marginalized individuals being targeted through arbitrarily enforced bylaws. One queer activist who has organized around issues of sting operations in public greenspace elaborated on this alternative approach:

It would be really great if there’s no police in parks, but then you need some kind of emergency, you know, people with healthcare training, for example, who were there on call or wandering around so there’s some way of accessing some help.

(Greenspace Activism Organization Employee)

Others had similar suggestions:

There are other ways of making that safe. When we look at defunding police maybe some of the money could go to much more sensitive managers of public spaces that could be kind of there to help people if something goes awry. They would be first aid trained, you know, have naloxone training or whatever like someone who is looking out for the health of the people using the space, rather than someone that’s looking at policing the space, I mean that would be a huge step forward. It seems to me in public spaces a way better use of the public purse than to have police ride through on their horses or just walk through. Those are scary. And with good reason, because you know they kill people, they hurt people and they imprison people.

(Politician)

[Removal of police presence] signals to queers that it’s okay to be here and then back it up by keeping the police away from there, and having someone that can, you know, somebody who is looking after the safety of everybody involved, but that’s not the police. And that’s traditionally not the police, it won’t be the police, …this is about safety of the people using the park, it’s not about policing the people in the park.

(Queer Activist)

When asked about who is responsible for promoting more equitable access to greenspace, and what that might look like, there was uncertainty among key informants. Informants with experience in planning operations emphasized the need for community collaboration. Yet, they argued that while community involvement is important in driving change, the responsibility is on the city to facilitate ideation and feedback processes that could build more inclusive greenspace environments. They encouraged management operations to embrace change, and incorporate iterative planning processes into park design and management:

I think it involves many different partners, it has to involve the community. Otherwise, you’re not going to really be creating truly accessible inclusive places for people. But a lot of it has to come from the city. They hold a lot of the power in public spaces, they write the rules. They divvy up the funding. And so there’s a lot of responsibility on the city to, I think, initiate some of these conversations. It’s important for community members and advocates to push on things, but ultimately, the city has to be the one to be flexible and courageous enough to try something new.

(Environmental Planner)

Other KIs from the activist community also emphasized the importance of having the public and those with lived experience involved in shaping decisions around greenspace use and management so that they are reflective of community needs:

Ultimately it belongs to us… It’s our space… so let us use it… And that’s a community decision, and I think the more local, in a sense, the better, because we know our own communities, and we know what we need. Let’s have more greenspace, let’s make space safe by keeping police out of it, and let’s sign it and make it safe, by the way it’s managed for everyone, including people that have nothing.

(Queer Activist)

Informants also shared thoughts on the institutional systems and policies that determine planning operations for greenspace in Toronto, citing that they often operate in discriminatory ways or lack transparency or clarity:

Systems that are in place that are often harmful and often possess several gaps which lead to the exclusion and often discriminatory development practices that we see today within the City of Toronto… For example, when talking about acknowledging that there are different genders that exist, and not just leaving it to woman or man or mom or father and stuff like that within policies. That needs to be more diverse and not just talking about the nuclear family because that’s a very outdated concept and the nuclear family is also something that is not really prominent in today’s times right? Families look different. Policy is just so behind… not only the language needs to be updated, but the concepts and ideas surrounding it as well.

(Urban Planner)

When you read the Provincial Policy Statement, for instance it’s a very interesting document in a way that it’s quite idealistic. First time I read it, it was just like oh my God, this is amazing, let’s implement it as written. And then you realize how this is translated into municipal policies in a way that is like whatever we’re not going to really pay attention, or we’re just going to pretend that we’re listening to it and not going to do anything about it… to have any effective change in any way. I know there’s a lot of people that are very critical to planning as a science as a whole, because at the end of the day, we’re just upholding status quo.

(Environmental Planner)

Overall, each key informant expressed the need for valuing community input, and building out the concept of accessibility within greenspace and environmental sustainability policy, planning, and management more broadly, to consider unique communities in their own rights, rather than adopting a top-down approach that narrowly serves presumed homogenous greenspace experiences and needs.

## 5. Discussion

The growing critical mass around the desire to build more sustainable urban spaces, along with broader ‘just’ sustainability movements and agendas [47], provide entry points for confronting contradictions within urban environmental and/or public space management, namely circumstances where practice is not representational, considerate of diversity and inclusion, or socially just [48]. Such circumstances misalign with several sustainable development pillars, including the goal of ‘providing good quality of life to all’ as outlined within the United Nations’ Agenda for Sustainable Development [49]. In other words, there is a growing understanding that there is no sustainability without a just and inclusive approach, as environmental quality and human equality are intertwined. Therefore, it is no longer acceptable for environmental practitioners to assume that issues of justice, equity and inclusion are beyond their mandate [47]. As such, the building of sustainable urban spaces must prioritize equitable access to social and environmental resources and amenities [5,6,47]. 

We contribute to these broader debates, and more specifically the field of sustainable greenspace management through novel integration of the concepts of environmental justice and moral management to reveal systemic barriers that impede access to urban greenspace and associated health benefits among 2SLGBTQ+ communities. Our work addresses the absence of attention to 2SLGBTQ+ communities across environmental justice scholarship broadly [7], and more specifically within studies of urban greenspace inequalities, despite public awareness of ongoing targeted violence, policing and exclusion in urban parks.

Further, queer ecology studies that examine nature and the environment through a queer lens have similarly not paid much attention to greenspace access as a justice issue [50], despite evidence of greenspace’s potential to support health, wellbeing and belonging in urban spaces [1]. We contribute to these gaps through exploring what is known about experiences of exclusion/access for queer communities in Toronto’s urban greenspace and whether and how these concerns are being addressed within urban greenspace and park management practice. 

Our results suggest that advancing inclusive and environmentally just greenspace for queer communities requires: (a) strengthening inclusion and further democratizing greenspace through environmental policy and practice; (b) reconsidering how to maintain public safety while reducing the criminalization of non-heteronormative users; and (c) signalling safe & inclusive spaces through symbolism and signage at the ground level. Our results also illustrate the concept of heteronormativity in greenspace, unveiling how the hegemony of heterosexuality is codified in Toronto greenspace, shaping what is accepted as appropriate behaviour/identities, and who gains inclusive access as a result. 

Greenspace is a critical site of interaction between private and public realms, which ultimately shape whose ideals and preferences get to inform decisions or become privileged within society [51]. The concept of moral management assists in revealing the ways in which the delineation of private and public spheres in greenspace places the personal in public. Expressions of gender and sexuality are regulated or conducted based on normative understandings of human nature and morality [52,53]. Unpacking how power and privilege are granted through the embodiment of normative identities divulges how some groups are privileged through interactions with and/or impacted from systems of greenspace [48] and environmental resources [47], while others are pushed to the margins. 

Greenspace access contributes to opportunities for 2SLGBTQ+ individuals and communities to live their lives fully, realize their sexual identities and contribute to their communities by being publicly visible. Greenspace additionally provides the spatial resources necessary for community gathering, cultural relations, leisure and the formation of emancipatory movements based on identity politics that transcend normative sexualities and binaried gender norms [54,55]. Yet, the promotion of public greenspace as democratized and open to all is disingenuous when social practice and systems limit participation, movement, and occupation of space at ground level [56].

Our findings demonstrate how hostility towards 2SLGBTQ+ occupants, discriminatory police patrolling in parks, institutional homophobia, and/or lack of acknowledgement of or attunement with 2SLGBTQ+ communities is creating access barriers to public greenspace among this community. Under the Municipal Code, it is instructed that those who violate bylaws are subject to removal from parks. Removal ultimately ends in displacement. This can be interpreted as an act of violence, where as a disenfranchised social group, 2SLGBTQ+ communities already experience ephemerality in spaces for gathering and leisure, in addition to limited access to natural amenities, health-supporting landscapes and safe social spaces, and may not have alternatives to turn to in the wake of removal [57]. 

In the policing of public space more broadly, urban geographers have revealed how marginalized individuals and groups are targeted by police utilizing punitive measures, resulting in charges and acts of reprimandation extending beyond what is necessary for the perceived threats to safety [58,59]. These practices of socio-spatial control call back notions found in moral geography, where moral meaning is a guiding principle for the management of public spaces such as greenspace through policing the behaviours of occupants by criminalization or fear of being reprimanded; or banishing them entirely [58,60]. The goals of removal and displacement act to make any subversion of normative life invisible in order to uphold the status quo and visual purity values, where non-normative visible identities or activities threaten the aesthetic of greenspace [26]. In terms of sexuality, strategies of control employed by police in greenspace purposely or implicitly link queerness to danger, or as a threat to the community, where in reality, as Robertson & McCleod describe, “being queer in public space is often the real danger: not queer people in public space.” [61]. Drawing from this vantage point, the findings suggest the central perceived ‘threat’ is when queer corporeality, kinship and love subvert deeply entrenched heteronormative social values and understandings of sexuality, partnership, gender, and domesticity. These visible identities and acts destabilize hegemonic cultural and institutional understandings of morality and daily life and thus need to be regulated, where the easiest path to achieve so is discouragement through criminalization, and ultimately, removal. Vague bylaws such as trespassing, loitering, and nuisances download responsibility onto individual police officers to interpret public (green)space occupants’ actions and intentions and cite and arrest them as they see fit. 

Utilizing laws to criminalize queer sexuality is not a new practice in policing. Police routinely conducted raids of bathhouses and other queer venues through the 1960s under the guise of different laws [57,62]. Homogenized institutional understandings and representations of greenspace in policy, or among managers and practitioners and/or the general public are rooted in normative understandings [63]. These understandings may be quite disparate from the actual lived experiences of various user populations. A limited scope can perpetuate an exclusive concept of “true space” where diverse, dynamic, competitive and embodied aspects of socially constructed greenspace use is unauthorized, unrecognized and/or not considered by dominant culture and therefore left out of management and planning practices [64]. 

Building inclusivity into greenspace planning exists at the upstream end of structural changes promoting health, as it targets a “distal cause of disease” [65]. This makes interventions somewhat subverted in the political realm, creating a barrier to the practical and timely development and implementation of policy promoting access and inclusion, especially in the absence of persistent community activism. Poor communication between stakeholders and the diversity of needs across neighbourhoods also contributes to this barrier, as equitable policy development and planning measures are complex [65]. A Toronto-based study by Newman [66] explored the work of non-profit organizations in encouraging planners to embrace diverse uses of greenspace like community gardens spaces and turn park landscapes into sites of reclamation. The findings concluded that community and equity-focused approaches to greenspace management, increased the potential for added complementary benefits including increased community food security, job creation and overall wellbeing for nearby residents. Recognizing that greenspace management is a multi-scalar issue and endeavour, community-driven approaches at the local park or greenspace level can additionally be supported by anti-gentrification policy development through a commitment to providing affordable housing, land trusts and rent stabilization programs [67]. Although these efforts require a careful balancing act, they have the potential to work cohesively to improve urban greenspace access, public health and social justice standings for 2SLGBTQ+ people and marginalized communities more broadly.

Cohesion and well-defined goals among planners, communities and stakeholders are integral in achieving a high standard for equitable greenspace access. However, what is more important than clear objectives is local politics [68]. Achieving equitable policy development in greenspace planning requires establishing a strong grassroots activism base and socially progressive politicians who can maximize the use of existing resources, strategically leverage current development processes, and see the value in a dynamic, socially equitable greenspace system.

In the case of greenspace access in Toronto, the othering of 2SLGBTQ+ people for threatening normative behaviour actively excludes this community from protection and forms a basis for socio-spatial control. Further, it limits 2SLGBTQ+ communities’ ability to access related greenspace benefits to health and wellbeing. As discussed by key informants, equitable access as a central goal to greenspace provision is not out of reach if institutional understandings of accessibility are holistic, well-researched, thoroughly defined and genuinely understood. 

We could move towards this goal through feasible action items including erecting visible queer flags, signage (or items of the like) in greenspace for symbolic representation. Reaching a desirable outcome could also require more complex changes, such as limiting policing and moving towards a community-driven model of ensuring safety in public parks. Greenspace management practitioners should look to develop holistic definitions of accessibility in order to create planning operatives and policy documents that better understand the interchange between 2SLGBTQ+ identities, physical access, safety, park upkeep and community contexts.

Our study does have limitations. This project is exploratory, with little existing analysis to build from. Consequently, this research provides a broader perspective on the state of greenspace accessibility for the 2SLGBTQ+ community in Toronto, and does not intend to paint a fulsome picture for each respective park space in Toronto, where 1473 parks are situated [44], each with their own unique social and physical features. Catungal and McCann [26] remind us of the importance of paying attention to context-specificity, arguing that park spaces should be studied in their own right, rather than approaching urban parks as monoliths that treat all marginalized groups in similar ways. Nonetheless, findings from this research can assist in providing a guiding framework towards more inclusive park management operations that can then be customized into context-specific forms for implementation at individual park levels in Toronto as we do not perceive or promote parks as homogeneous spaces. Moving forward the 2SLGBTQ+ community must be engaged explicitly by policymakers, planners and scholars to address the subvert processes leading to inequitable access to nature in the city and to develop appropriate, geographically specific solutions. The 2SLGBTQ+ community in Toronto includes a diversity of identities, experiences, values, worldviews and beliefs. This research, at times, presents this community in an analogous fashion. While accounting for nuances is a priority, it is also necessary to normalize some likeness across the queer community in order to express findings in a way that is useful and promotes kinship for the benefit of the community overall. Defining a subject population is an arduous task when researching the perceptions of a community whose culture includes the rejection of titles and binaries and is both bounded and fluid in identity expression and embodiment. Our original intention was not to adopt purely a queer stakeholder lens, however among the key informants involved in greenspace management that were willing to participate, all identified as queer. This suggests knowledge and awareness on this issue is largely held intra-communally, as non-2SLGBTQ+ participants either did not respond or indicated that this issue was outside of the mandate of their job or expertise (an interesting finding in and of itself).

We acknowledge that some of the insights raised by our study participants may not have been as apparent among other key greenspace stakeholders that do not have lived experience identifying as a 2SLGBTQ+ person. Further, we recognize there remains room to explore greenspace inequities within this community from a more intersectional perspective, that considers other interactive factors such as the role of race, age, socioeconomic status, etc.

## 6. Conclusions

This research provides extensive insight into the complexity of urban parks as political spaces and the spectrum of ideologies around greenspace in cities and whom they are for. While acknowledging that public safety is of utmost importance, assumptions about who belongs in the public realm and who deserves protection must be engaged with critically. Criminalization and violence towards 2SLGBTQ+ communities in urban parks motivated this inquiry into how normative framings of urban greenspace are reproduced and enforced through greenspace management, governance, and regulation. Considering the notable amount of media coverage on 2SLGBTQ+ targeted violence and police operations in greenspace, the lack of understanding of access barriers is concerning and reveals a significant gap in scholarly literature. 

The ongoing failure to recognize and act to resolve known management issues over non-normative sexual identities in greenspace makes Toronto’s branding and associated goals to be a sustainable and inclusive city pious and disingenuous. Most importantly, leaving this issue unexamined robs 2SLGBTQ+ individuals of dignity and agency over their lifestyles and personal expression, while advancing environmental injustice, unwarranted criminalization, isolation, and a lack of public access and rights to the city. Urban greenspace is a dynamic, diverse social system that requires ongoing management that not only acknowledges but celebrates its wide array of users and uses by working towards safe, equitable access for all. 

## Figures and Tables

**Table 1:** Key municipal documents analyzed.

Key Documents Analyzed
*Toronto Parks Plan* (2013)
*Planning Act* (2018)
*Toronto Parks and Recreation Facilities Master Plan* (2017)
*City of Toronto Parkland Strategy (Final Report)* (2019)
*City of Toronto Municipal Code Chapter 608: Parks* (2018)

## Data Availability

Not applicable.
